# Direct domain adaptation through reciprocal linear transformations

**DOI:** 10.3389/frai.2022.927676

**Published:** 2022-08-11

**Authors:** Tariq Alkhalifah, Oleg Ovcharenko

**Affiliations:** Physical Sciences and Engineering, King Abdullah University of Science and Technology, Thuwal, Saudi Arabia

**Keywords:** domain adaptation, covariate shift, supervised learning, MNIST classification, deep learning

## Abstract

We propose a direct domain adaptation (DDA) approach to enrich the training of supervised neural networks on synthetic data by features from real-world data. The process involves a series of linear operations on the input features to the NN model, whether they are from the source or target distributions, as follows: (1) A cross-correlation of the input data (i.e., images) with a randomly picked sample pixel (or pixels) of all images from the input or the mean of all randomly picked sample pixel (or pixels) of all input images. (2) The convolution of the resulting data with the mean of the autocorrelated input images from the other domain. In the training stage, as expected, the input images are from the source distribution, and the mean of auto-correlated images are evaluated from the target distribution. In the inference/application stage, the input images are from the target distribution, and the mean of auto-correlated images are evaluated from the source distribution. The proposed method only manipulates the data from the source and target domains and does not explicitly interfere with the training workflow and network architecture. An application that includes training a convolutional neural network on the MNIST dataset and testing the network on the MNIST-M dataset achieves a 70% accuracy on the test data. A principal component analysis (PCA), as well as t-SNE, shows that the input features from the source and target domains, after the proposed direct transformations, share similar properties along the principal components as compared to the original MNIST and MNIST-M input features.

## 1. Introduction

Machine learning (ML) is gaining a lot of traction as a tool to help us solve outstanding problems in image processing, classification, segmentation, among many other tasks. Most of the applications in many fields have relied on supervised training of neural network (NN) models, where the labels (answers) are available (Ronneberger et al., 2015; He et al., 2016; Osisanwo et al., 2017; Di and AlRegib, 2020). These answers are often available for synthetically generated data as we numerically control the experiment, or they are determined using human interpretation or human crafted algorithms applied to real data. The challenge in training our NN models on synthetic data is the generalization of the trained models on real data, as that process requires careful construction of the training set and the inclusion of realistic noise and other features from the real data. In other words, the synthetic and real data are usually far from being drawn from the same distribution, which is essential for the success of a trained NN model (Kouw, 2018). Thus, many synthetically trained NN models have performed poorly on real data. On the other hand, training on real data using supervised learning provides models that are often, at best, as good as the accuracy of the labels that where determined by humans or human crafted algorithms (weak supervision). So the data-driven feature of machine learning, in this case, will be highly weakened (Zhou, 2017).

### 1.1. Related work

The concept of trying to bridge the gap between the distributions of the training (source) and application (target) data is referred to as domain adaptation (Kouw, 2018; Lemberger and Panico, 2020). In this case, the training dataset is assumed to belong to the source domain and the application/testing data are assumed to belong to the target domain, the target of our training. The classic theory of machine learning assumes that the application (target) data of a trained model come from the same general population (sampled from the same distribution) as the training (source) set (Kouw and Loog, 2019). So we need the probability distribution of the synthetic (source) dataset, *P*_*s*_(**x_s_**, **y_s_**), where **x_s_** are the input features, and **y_s_** are the labels from the source domain, to be similar to the probability distribution of the real (target) dataset, *P*_*t*_(**x_t_**, **y_t_**), where **x_t_** are the input features, and **y_t_** are the labels for the target set. In real life, target labels are often missing or are very limited, and thus, we have to assume that the training task is representative of the application (inference) task. In other words, the synthetically modeled labels reflect what we would expect in real life granted that the input features are similar. However, though the task might be well-represented by the training data, like a classification of a picture of a “cat” as a cat, and such data might be generated through computer simulation (of a cat), the application data probably based on real pictures might have slightly different features (i.e., background) as compared to the training (source) data. One category of data adaptation is referred to as subspace mapping (or more generally, alignment) in which we find a transformation, *T*, that results in the distribution of the training (source) input features to equal that of the target data (Fernando et al., 2013; Kouw et al., 2016). Specifically, *P*_*s*_(*T*(**x_s_**)) = *P*_*t*_(**x_t_**). This can, also, be accomplished by projecting the source and target input features to the eigenvectors of the two subspaces, then finding a transformation between these projected spaces. Such projections can be achieved by Neural network embedding, in which we find the weights of the embedding that minimizes the distance between the distribution of the source samples embedding and the target samples one. There are many ways to find the transformation or weights to make the distributions similar including the use of optimal transport (Villani, 2008). Training data selection through a reinforcement learning framework can help in domain adaptation by employing training instances relevant to the target domain (Liu et al., 2019). Even cycle generative Adversarial networks (GANs) are used for the purpose of learning a generator to map target input features to source ones (Ganin et al., 2016). However, these methods become more difficult to apply when the dimensions of the input features are large. The method, proposed in this paper, shares the general concept of this objective implemented in a direct fashion.

### 1.2. The paper's objectives

An objective of a trained neural network model is to provide us with an output for a given input. The output that a trained model will give is based on the training it experienced, and that depends mostly on the source training set and its distribution. A trained neural network model generalizes well when the target data are represented, as much as possible, in the source data set. To help accomplish that when the application (target) data are available, we propose, here, to inject the real data features into the synthetic data training. This can be accomplished by utilizing a combination of linear operations including cross-correlation, auto-correlation and convolution between the source and target input features. These operations will bring the distributions of the training (source) input features, and the testing (target) input features closer to each other, which will help the trained model generalize better in the inference stage. In this paper:

- We propose a new transformation of the input features based on linear operations that will allow us to bring their distributions closer; we refer to this explicit process as direct domain adaptation (DDA).
- The process is explicit and it operates exclusively in the data domain, without intervention into the training workflow and architecture. The method achieves 70% accuracy when a relatively simple model is trained on the MNIST data and applied to the MNIST-M data.
- We use principal component analysis (PCA) to show how much the principal components of the MNIST and MNIST-M input features after DDA came closer to each other.

The paper is organized as follows. We first describe the setup of the problem and our scope, and then share the proposed linear transformations. We then share the results of testing the approach on the MNIST/MNIST-M data. Finally, we discuss the effect of the transformation on the data, and share some concluding remarks.

## 2. Input features reciprocal projections

To bring the distributions of the input features of the source and target data closer, we apply linear transformations that will allow some of the critical information, like background, of the inputs to migrate between the two data without effecting the key features needed for the task at hand. After transformation, the input features will have two instances of each data (source and target) embedded in them. The method proposed here is applicable mainly to supervised and in some cases of unsupervised (i.e., clustering) learning. We will focus here on supervised learning, and specifically classification. We will first share the setup of the problem and then describe, in details, the proposed transformations.

### 2.1. Setup

For the neural network (NN) model, we consider the input feature space χ, a subset of *R*^*d*^, where *d* is the dimension of the input. We also consider the label space *Y*, a subset of *R*^*D*^, where *D* is the dimension of the output. We assume we do not have labels in the target domain, and thus, we can not perform transfer learning (a form of domain adaptation Hanneke and Kpotufe, 2019). So in our case, the source data are labeled, but the target real data are not. Thus, we consider sample source data having inputs **x_s_** and labels **y_s_**, having a probability distribution *P*_*s*_(**y_s_**, **x_s_**), that is different from the target data with inputs **x_t_** and potential labels **y_t_**, given by a probability distribution *P*_*t*_(**y_t_**, **x_t_**). We assume that the distribution difference is caused by the shift in the input features, and thus, *P*_*s*_(**y_s_**|**x_s_**) = *P*_*t*_(**y_t_**|**x_t_**). This sort of difference between the source and target datasets distributions is referred to as a covariate shift (Fernando et al., 2013). In this case, the issue we are addressing here is the case when the input (features) distributions for the source and target data are not the same, specifically *P*_*s*_(**x_s_**) ≠ *P*_*t*_(**x_t_**).

There are many ways to measure such a shift, including using the Kullback-Leibler (KL) divergence metric. In domain adaptation, and similar to error bounds defined for machine learning in general, we can define an error bound on the application of a trained network model. This error bound is given by the error in the training and a term related to the complexity of the NN model (like its size). This, however, assumes that the training and application data come from the same distribution. For the case of a covariate shift, we have a similar bound, given by (Ben-David et al., 2006; Lemberger and Panico, 2020):


(1) $$\begin{array}{l} \varepsilon_{t}(NN)\leq \varepsilon_{s}(NN)+dist(P_{s}({\text{x}}_{\text{s}}),P_{t}({\text{x}}_{\text{t}}))+\text{λ}, \end{array}$$

where ε_*s*_ is the bound on the training error, and *dist*(., .) is the distance between the marginal distributions of source and target input features. Here, λ represents the optimal joint errors of the neural network model between the source and target datasets. So the upper bound of the application error is guided by these three terms.

### 2.2. Our goal

One category of data adaptation is referred to as subspace mapping (or more generally, alignment) in which we find a transformation, *T*, that results in the distribution of the training source input features to approximate that of the testing ones (Fernando et al., 2013). Specifically, *P*_*s*_ (*T*(**x_s_**)) ≈ *P*_*t*_(**x_t_**). Compared to previous transformations, the approach used here does not require any eigenvalues computation, which can be expensive for large dimensional input features.

**Figure 1:**
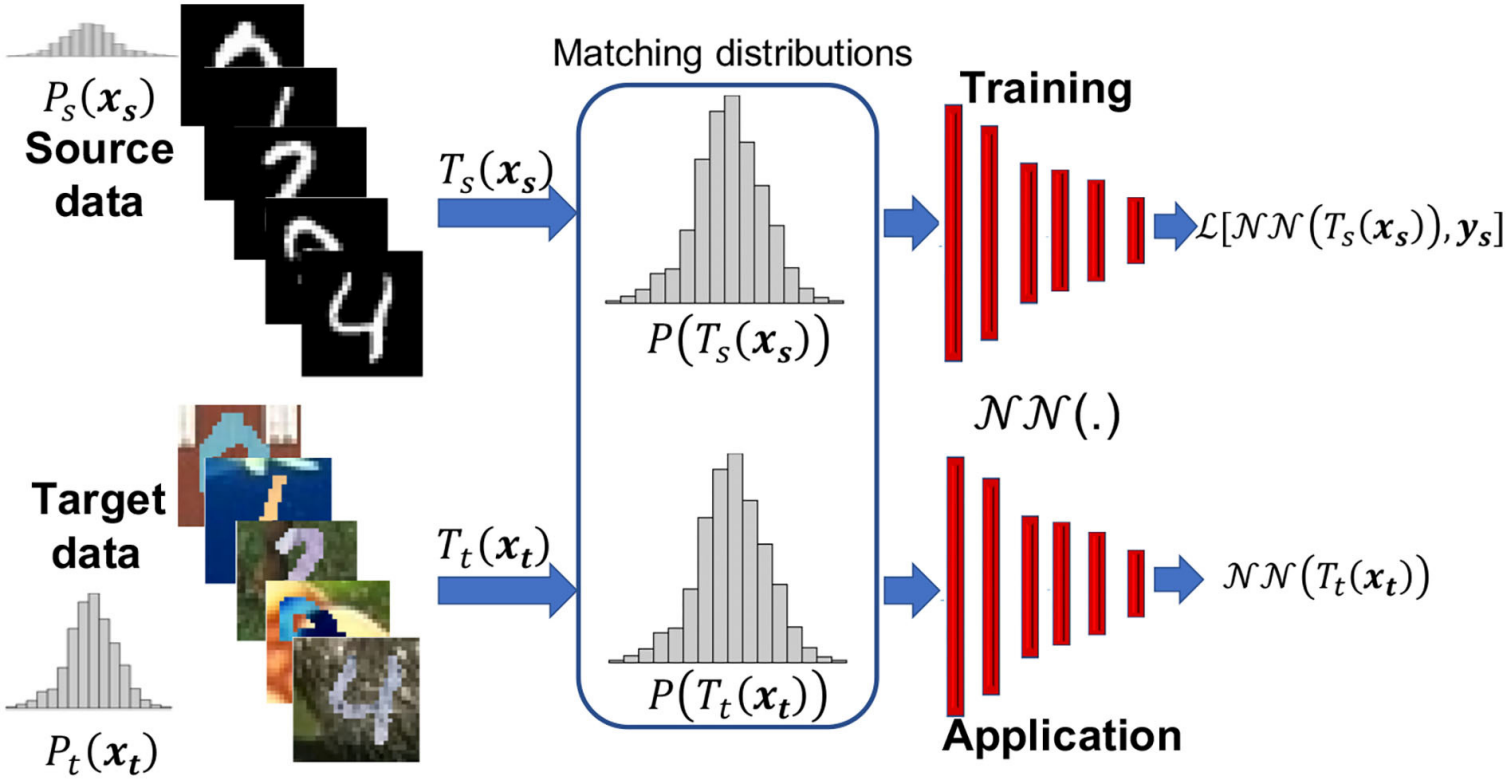
The motivation behind the proposed data adaptation in which the training (source) dataset might have a different distribution than the application (target) dataset, in which transformations *T*_*s*_ and *T*_*t*_ will help reduce such difference and provide new input features to the neural network function (NN) to train the network to reduce loss L, and then apply to real data. Here, *P* is the probability distribution and we show schematic versions of it for the source and target data, given by samples of the MNIST and MNIST-M datasets, respectively.

So our objective is to devise a transformation that minimizes the difference measure *d* in Equation (1) between the distributions of the training (source) and testing/application (target) input features. Specifically, we aim to find the transformation ${\bar{x}}_{\text{s}}=T_{s}({\text{x}}_{\text{s}})$ on the source input features and ${\bar{x}}_{\text{t}}=T_{t}({\text{x}}_{\text{t}})$ on the target input features so that the probability distributions $P_{s}({\bar{x}}_{\text{s}})\approx P_{t}({\bar{x}}_{\text{t}})$. Figure 1 summaries that within the framework of the training process. So the input to the training of the Neural network (NN) model is ${\bar{x}}_{\text{s}}$, in which the model parameters are optimized to match the labels **y_s_** using a loss function (L). As our focus are the input features and their transformation, the labels, **y_s_**, for training remain the same, and thus, the loss function used here is the classic one needed for the task at hand, which for classification, as an example, would be the cross entropy. The loss would be measured between the prediction and **y_s_**. On the other hand, the input during inference is ${\bar{x}}_{\text{t}}$. We will discuss the transformations *T*_*s*_ and *T*_*t*_ in the next subsection.

### 2.3. Transforming the input features

Without loss of generality, we focus our development on two-dimensional input features, like images. The approach is applicable to one or three dimensional input features by using the corresponding one or three dimensional convolution and correlation operations.

Images in general, including pictures and medical scans, can often be represented by a combination of reflectively, **r**, source (like source of light), **s**, and noise **n**, as follows:


(2) $$\begin{array}{l} {\text{x}}_{\text{t}}^{\text{i}}={\text{r}}^{\text{i}}*\;{\text{s}}^{\text{i}}+{\text{n}}^{\text{i}}. \end{array}$$

Depending on the data, all three components (**r**, **s**, **n**) can vary over input target features (indexed by **i**), and have their own distributions. For ML training, we often use synthetically generated images, where the labels are known, considering the data are more likely simulated. To help improve the generalization of such trained NN models on images represented by Equation (2), we often try to include a proper representation of these components from the target input features into the synthetic (source) input features. However, in many cases, this is hard and costly. and that will effect the generalization of the trained model. For one, our synthetic data are often free of noise or we only add random noise to them. So we propose here operations that will transform information between the source and target input features.

To migrate the components described in Equation (2) from the target input data to the source, we use the following linear operations to obtain the transformed input source features:


(3) $$\begin{array}{l} {\bar{x}}_{\text{s}}^{\text{i}}=T_{s}({\text{x}}_{\text{s}}^{\text{i}})={\text{c}}_{\text{s}}^{\text{i}}({\text{x}}_{\text{s}}^{\text{i}})*{\bar{a}}_{\text{t}}({\text{x}}_{\text{t}}), \end{array}$$

given as a convolution of representations from the source, ${\text{c}}_{\text{s}}^{\text{i}}$, and the target input features ${\text{c}}_{\text{s}}$. Specifically, to properly scale the input source features for training, we correlate them with randomly drawn pixel (or pixels) values, ${\tilde{x}}_{s}^{j}$, from input source image indexed by *j*, as follows:


(4) $$\begin{array}{l} {\text{c}}_{\text{s}}^{\text{i}}({\text{x}}_{\text{s}}^{\text{i}})={\text{x}}_{\text{s}}^{\text{i}}⊗{\tilde{x}}_{s}^{j}. \end{array}$$

The random index *j* varies per input image and per epoch to allow for proper representation of the pixels in the training in which the input images are correlated, with operator ⊗ representing cross-correlation. This operation amounts to scaling the input features with representations of the source domain. The output from this operation is then convolved with the mean of the auto-correlated target data input features, given by


(5) $$\begin{array}{l} {\bar{a}}_{\text{t}}({\text{x}}_{\text{t}})=\frac{1}{N_{t}}\sum_{\text{j}}^{N_{t}}{\text{x}}_{\text{t}}^{\text{j}}⊗{\text{x}}_{\text{t}}^{\text{j}}, \end{array}$$

where *N*_*t*_ is the number of input features (images) from the target domain. The auto-correlation here, considering 2D input features, is a two-dimensional one and is applied in the Fourier domain, along with the convolution. In the Fourier domain, these operations reduce to simple multiplications. The mean of the auto-correlation of the input features provides a measure of the distribution of the data. For example, the auto-correlation of random noise yields a quasi delta-function at zero lag proportional to the energy of the noise. A convolution with such a function will incorporate that energy into the synthetic data so that the signal-to-noise ratio (SNR) in the transformed source input features would be comparable to that of the auto correlated target data. To allow for the transformed input features from the target domain to have a similar distribution to the transformed input features from the source domain, we apply a similar transformation to that in Equation (3) with the roles reversed.

**Figure 2:**
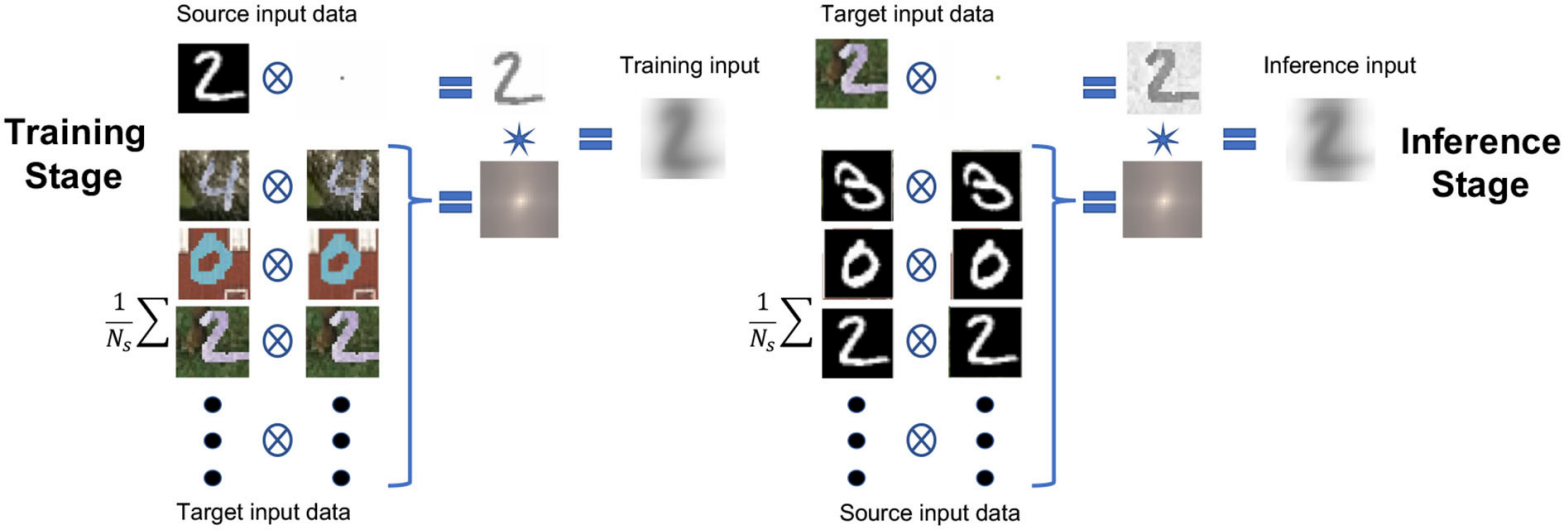
The workflow chart for the proposed data adaptation applied as an example to the MNIST/MNIST-M datasets. On the left, the proposed process used for producing the training data; on the right, the proposed process used for producing the testing/application data. The circled cross symbol denotes a cross-correlation operation, and the star symbol denotes a convolution operation.

As such, we apply the following transformation on the target input features during the inference stage:


(6) $$\begin{array}{l} {\bar{x}}_{\text{t}}^{\text{i}}=T_{t}({\text{x}}_{\text{t}}^{\text{i}})={\text{c}}_{\text{t}}^{\text{i}}({\text{x}}_{\text{t}}^{\text{i}})*{\bar{a}}_{\text{s}}({\text{x}}_{\text{s}}), \end{array}$$

which includes the same operations as in Equation (3) with the role of the source and target input features reversed. Thus,


(7) $$\begin{array}{l} {\text{c}}_{\text{t}}^{\text{i}}({\text{x}}_{\text{t}}^{\text{i}})={\text{x}}_{\text{t}}^{\text{i}}⊗{\tilde{x}}_{t}={\text{x}}_{\text{t}}^{\text{i}}⊗\frac{1}{N_{t}}\sum_{\text{j}}^{N_{t}}{\hat{x}}_{t}^{j}, \end{array}$$

where, now, ${\hat{x}}_{t}^{j}$ represent randomly drawn sample pixel (or pixels) from the input features from the target data to scale them with the mean, ${\tilde{x}}_{t}$. Also, the mean of the auto-correlation of the input features from the source data is given by


(8) $$\begin{array}{l} {\bar{a}}_{\text{s}}({\text{x}}_{\text{s}})=\frac{1}{N_{s}}\sum_{\text{j}}^{N_{s}}{\text{x}}_{\text{s}}^{\text{j}}⊗{\text{x}}_{\text{s}}^{\text{j}}, \end{array}$$

where *N*_*s*_ represents the total number of input features (images) from the source domain. The idea behind having two instances of the input features from each domain in Equations (4), (5), (7), and (8), is to balance their contribution in the resulting new source and target distributions. This way, we match properties of the input features from the source and target domains used for training and inference, respectively, and yet maintain the critical-for-classification features of the original input mainly intact. Note that the convolution operation that connects equal amounts of the source and target contributions in Equations (3) and (6) is commutative. We will see the value of this operation more in the next section. Meanwhile, Figure 2 (left) demonstrates the process of applying Equation (3), where the input features correspond to the MNIST original labeled data. On other hand, Figure 2 (right) demonstrates the process of applying Equation (3) on the MNIST-M data, which we assume to be label-free (the labels used for accuracy assessment). Note that the resulting images after transformation of the same digit look similar for the two processes. Using principal component analysis (PCA) and t-SNE, we demonstrate later how much these proposed transformations brought the distributions (at least their principle components) closer to each other. Since this approach is relatively direct, requiring no optimization or eigenvalue projections, we refer to it as direct domain adaptation (DDA).

The operations involved in DDA given by Equations (3) and (6) are designed to reciprocally embed the spectral features of one domain into the other, where the transformed data have equal instances of both data. The auto-correlation operation yields zero phase so that the phase information of each data (holding most the features needed for classification) remains intact. These features will be demonstrated in our Fourier analysis.

### 2.4. A Fourier domain analysis

If we transform the target images to the Fourier domain, considering the basic laws of the Fourier representation of cross-correlation and convolution, Equation (2) can be written as


(9) $$\begin{array}{l} X_{t}^{i}=A^{i}e^{I\phi^{i}}=R^{i}S^{i}+N^{i}, \end{array}$$

where *A* and ϕ are the amplitude and phase, respectively, of the complex-valued Fourier representation, with *I* representing the imaginary unit. All capital letters indicate the Fourier representation form of the reflectivity, source, and noise functions in Equation (9), given respectively. As a result, we can write Equation (3) in the Fourier domain as


(10) $$\begin{array}{l} X_{s}^{i}={\bar{X}}_{s}^{i}{\tilde{X}}_{s}^{j}(\frac{1}{N_{t}}\sum_{j}{\bar{X}}^{j}X^{j})={\bar{X}}_{s}^{i}{\tilde{X}}_{s}^{j}(\frac{1}{N_{t}}\sum_{j}{\left(A^{j}\right)}^{2}),\\ \;={\bar{X}}_{s}^{i}{\tilde{X}}_{s}^{j}(\frac{1}{N_{t}}\sum_{j}(R^{j}S^{j}+N^{j})({\bar{R}}^{j}{\bar{S}}^{j}+{\bar{N}}^{j})), \end{array}$$

where the overstrike, $\bar{.}$, symbol here stands for the complex conjugate. Note that the transformed source input features will contain elements of the target noise and reflectivity. More importantly these elements do not affect the shape of the features as they have zero phase.

The application of the model on target data will involve an input to the model given by Equation (6), which can be represented in the Fourier domain by


(11) $$\begin{array}{l} {\bar{X}}_{t}^{i}={\bar{X}}^{i}(\frac{1}{N_{t}}\sum_{j}{\hat{X}}^{j})(\frac{1}{N_{s}}\sum_{j}{\bar{X}}_{s}^{j}X_{s}^{j})\\ \;=({\bar{R}}^{i}{\bar{S}}^{i}+{\bar{N}}^{i})(\frac{1}{N_{t}}\sum_{j}^{N_{t}}({\hat{R}}^{j}{\hat{S}}^{j}+{\hat{N}}^{j}))(\frac{1}{N_{s}}\sum_{j}{\bar{X}}_{s}^{j}X_{s}^{j}), \end{array}$$

where $\hat{R}$, Ŝ, and $\hat{N}$ correspond to the aforementioned random pixel reflectivity, source, and noise, respectively, expressed in the Fourier domain. In this domain, for a single random pixel case, these quantities are constant, and the summation will render an average over the target input features. If the random pixel value is placed in the middle of the image as we do here, then all these quantities are real admitting no phase shifts, like in the case of the auto-correlation in Equation (10). So from Equations (10) and (11), we clearly notice that DAA given by both transformed input features contain equal representations of the source, reflectively and noise from the target domain. Since, the two data sets, after transformation, share the same elements sampled from their corresponding distributions, then the distributions of the two data sets should be close, which allows us to satisfy the requirement *P*_*s*_(*T*_*s*_(**x**)) ≈ *P*_*s*_(*T*_*s*_(**r**)) for the generalization of the NN model. In other words, the second term in Equation (1) will be small.

## 3. The MNIST data example

The MNIST dataset has commonly been used to evaluate the performance of supervised learning algorithms tasked to classify images (LeCun et al., 1998). The image dataset contains 60,000 training samples and 10,000 testing samples of handwritten digits, with their corresponding labels (the digits). Training accuracy has reached almost 100% (Byerly et al., 2021). As a follow up, the MNIST-M dataset was developed as a more complicated form of MNIST. The images of the MNIST digits were combined with patches randomly extracted from color photos, and thus, have 3 color channels rather than monochrome images in the MNIST dataset. The MNIST-M dataset has been used to test newly proposed domain adaptation methods (Long et al., 2015; Ganin et al., 2016). It is made up of 59,000 training images and 9,000 testing images, which we assume to be label-less.

### 3.1. The setup

As mentioned above, the target MNIST-M input features are colored images described over 3 RGB channels, whereas the available training data inputs are gray-scale. We transfer features of source dataset to the target dataset, and vice-versa, by a sequence of linear operations explained earlier. The transformed source dataset then is used for training of the network while the validation happens on the transformed target dataset. Since the MNIST data are single channel gray-scale images, we simply replicate the mono-channel values into the three channels.

**Figure 3:**
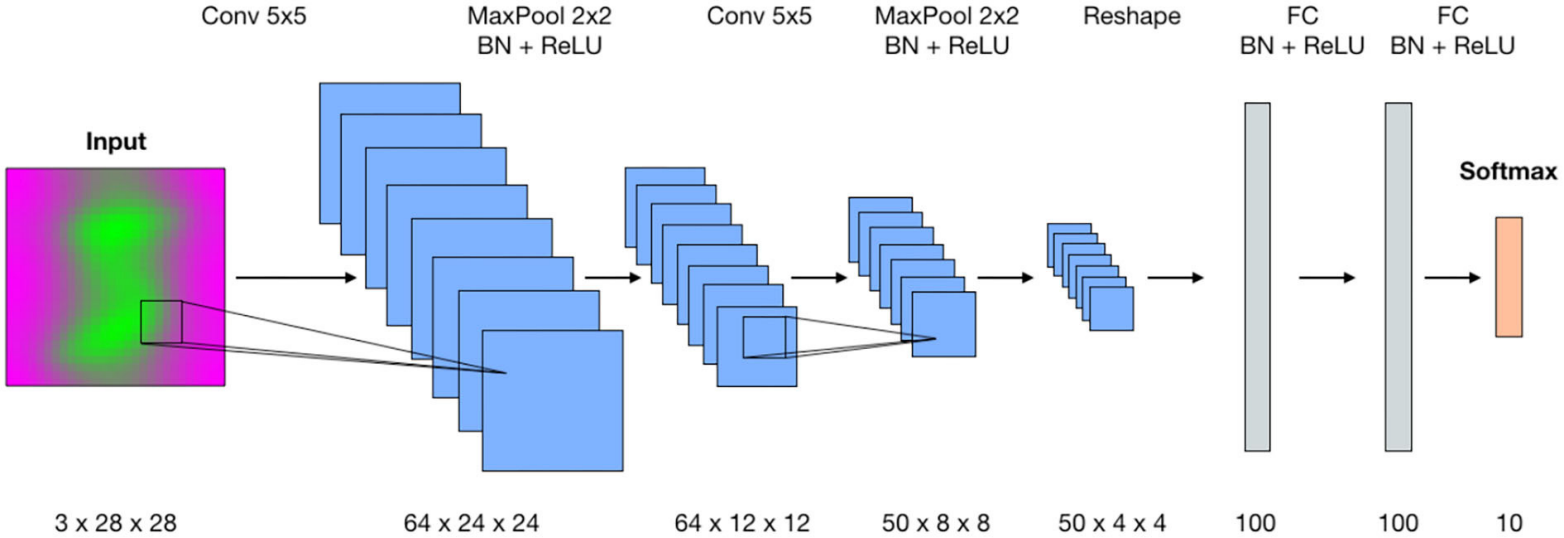
The architecture of the convolutional neural network for digit classification. The proposed method operates in the data domain and does not require intervention into the existing training workflow.

The architecture of the deep neural network for digit classification is inherited from Ganin et al. (2016). In this paper, the authors introduce the concept of domain adversarial neural networks for domain adaptation and design the generative-adversarial architecture (DANN) for this purpose. The generator used in their digit recognition example originally produces two outputs—categorical for digit labels and binary to identify the domain of the input data. We modify this generator by keeping only categorical output, which assigns labels from 0 to 9 for the input images. The resulting convolutional network (Figure 3) is a variant of the classic LeNet-5 architecture for digit recognition (LeCun et al., 1998). Specifically, the convolutional part of the network is built as a stack two blocks where each block consists of a convolutional layer, batch normalization and max pooling, followed by a ReLU activation function. The classification head is built as a set of fully-connected layers and batch normalizations followed by ReLU. The output from the last fully-connected layer is passed through the Softmax activation function that is equivalent to assigning probabilities among categorical labels. There are also two Dropout layers placed in the encoder and classification head.

### 3.2. Adaptation process

**Figure 4:**
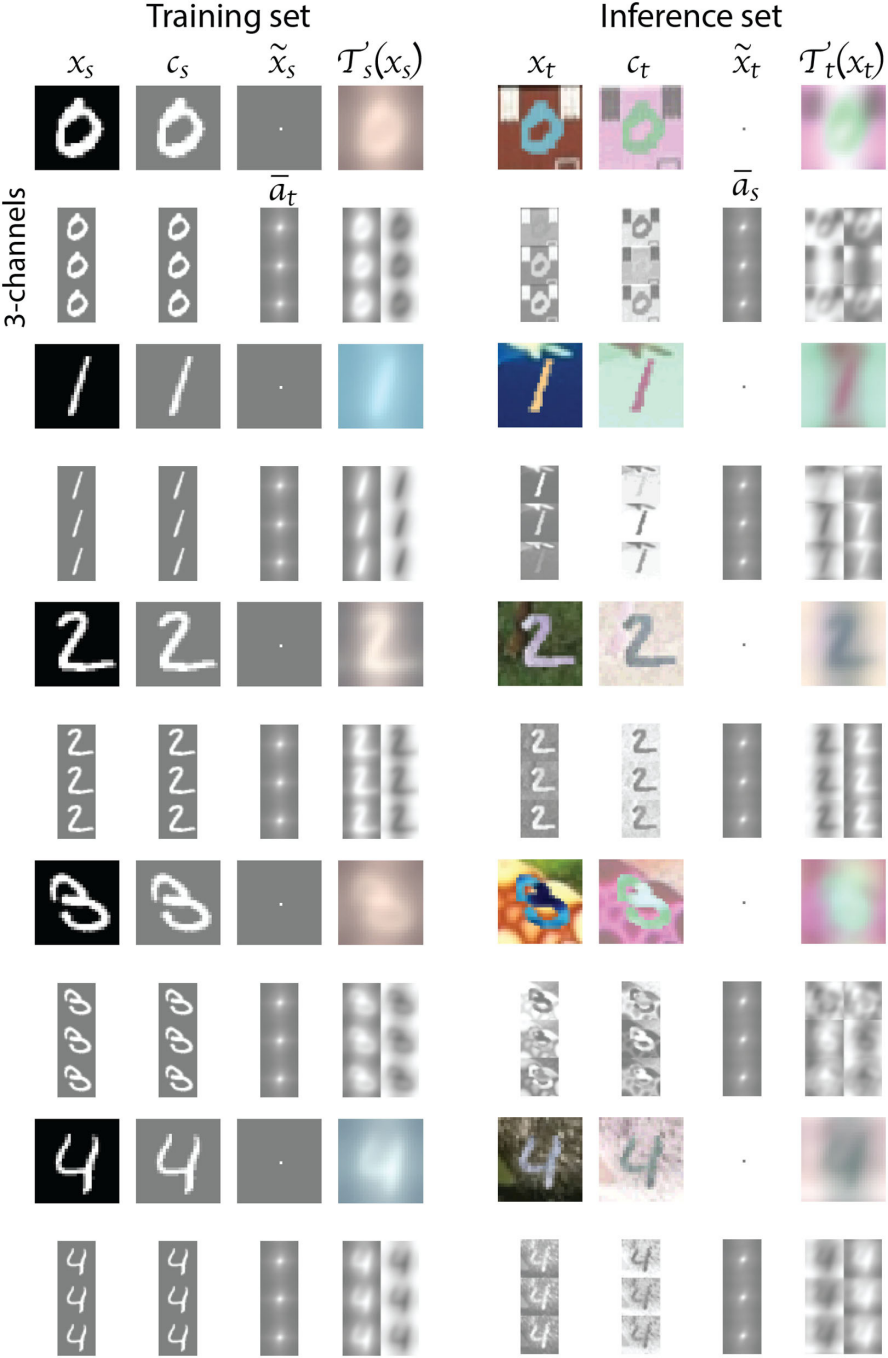
The transformation results for examples from digits 0 to 4 images. The left column shows the transformation of source (MNIST) data, and the right column shows the transformation of the target (MNIST-M) data. For each input, we show the composite and the 3-channel transformation. The last column of the second row shows the 3-channel output and its polarity reversal, which we implement in the augmentation of the training data.

**Figure 5:**
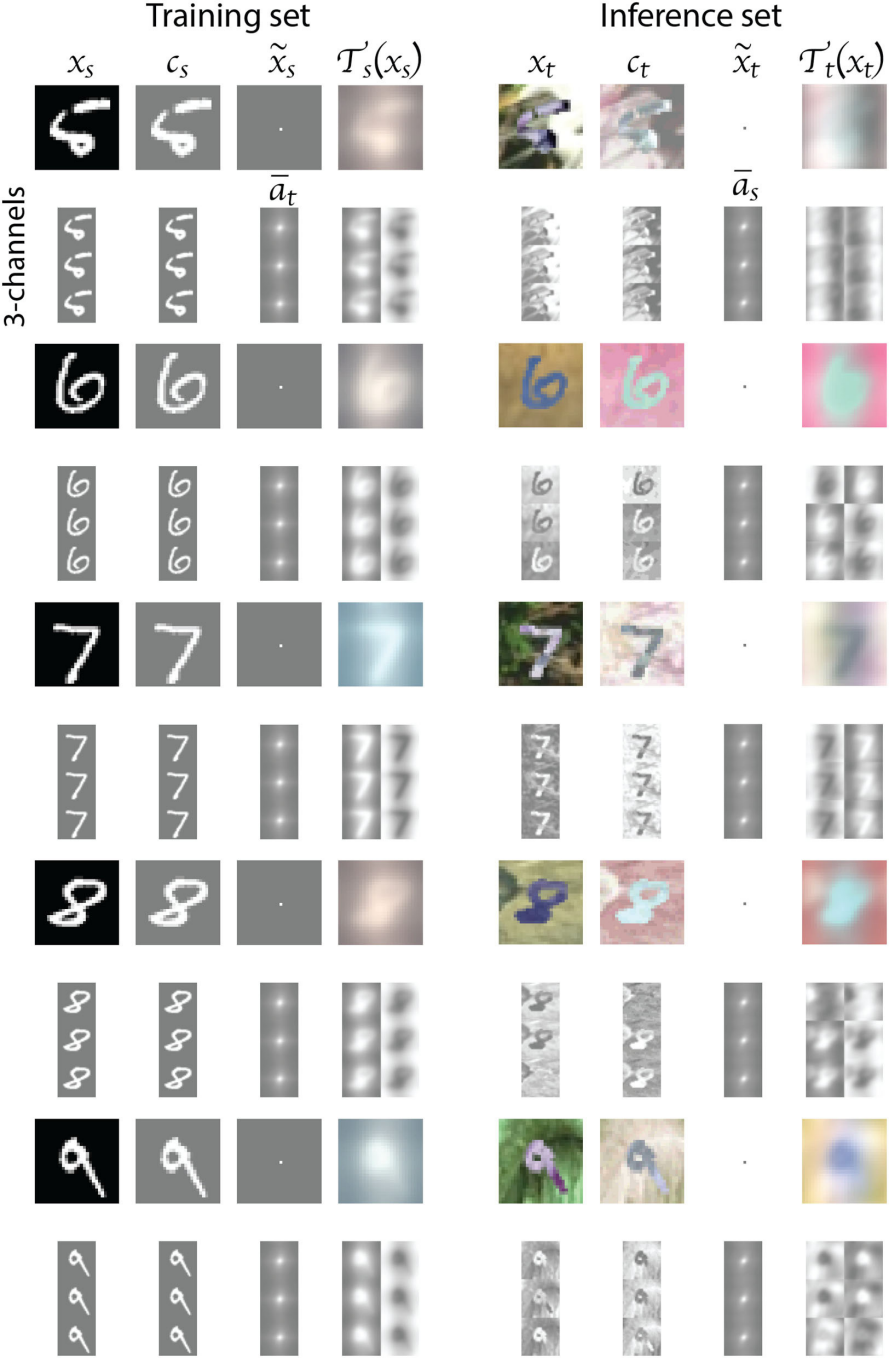
The transformation results for examples from digits 5 to 9 images. The left column shows the transformation of source (MNIST) data, and the right column shows the transformation of the target (MNIST-M) data. For each input, we show the composite and the 3-channel transformation. The last column of the second row shows the 3-channel output and its polarity reversal, which we implement in the augmentation of the training data.

We apply the DDA to the MNIST and MNIST-M data using Equations (3) and (6), respectively. In Figures 4, 5, we show the effect of DDA on an example image for each of the 10 digit classes. The left column of each Figure shows the training (source) samples transformation, while the right column shows the inference (target) samples. For each digit, we show the color image (top row), and the corresponding individual RGB channels (bottom row), which constitute the actual input to the classification network. The images labeled **c_s_**, **c_t_**, ${\bar{a}}_{\text{s}}$, and ${\bar{a}}_{\text{t}}$ correspond to Equations (4), (7), (5), and (8), respectively. Note that the final transformed input features for the source and target data look very similar. This similarity is more profound in the 3-channel representation, which are the actual inputs. We also, however, ended up with a reverse in polarity apparent in the input features for digits 0, 3, 5, and 8. This will be covered by the polarity reversal we will implement to augment the training (source) data, and such a reversal is demonstrated in Figure 4, as well.

### 3.3. Data augmentation and training

We apply DDA to the input training data on the fly. Specifically, given a sample of input data from the source (grayscale image), we first cross-correlate it with a random pixel drawn from another arbitrary sample from the same dataset. Then, we convolve the result with the mean of the auto-correlation of the entire target dataset and re-normalize the outcome to fit the [−1, 1] range. The re-normalization is trivial and implies subtracting from the input features the minimum value among all channels, then dividing by the maximum of the previous result, which will bound the values between zero and one, and finally we multiply the output by 2 and subtract 1.

At the inference stage, given a sample from the target dataset, we cross-correlate it with the average of pixel values from the same dataset (specifically, we randomly draw a pixel from each image in the dataset and compute their average) and convolve the result with the average of auto-correlation of the source dataset. For inference on a sample from source dataset for testing, we would reverse the domains and first cross-correlate the sample with the mean of pixel values from the source dataset, followed by the convolution with the mean auto-correlation of the target dataset.

We train the network for 100 epochs using a batch size of 128 and an Adam optimizer (Kingma and Ba, 2014) with the learning rate set to 1*e*−3. To emphasize the capability of our proposed DDA approach, we augment the data using trivial methods. These include random polarity reversal of color channels and channel-shuffling. The first one compensates for polarity mismatch between transformed images in the two datasets so we train the network to deal with it. The second augmentation effectively expands the dataset by changing the order of channels in the image.

**Figure 6:**
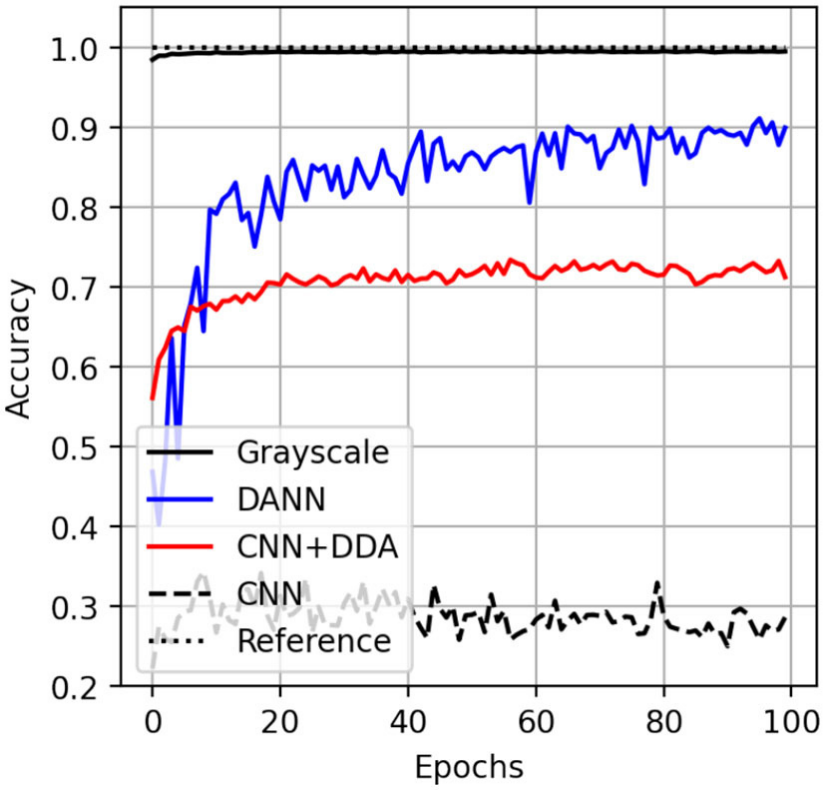
The accuracy curves for application of trained networks on test partition of MNIST (solid black) and MNIST-M (other) datasets. The inference score on MNIST-M by CNN (dashed), DANN (blue) and proposed method (CNN + DDA, red). The perfect score is shown as a dotted line at 1.

The inference accuracy on the transformed test partition of the source dataset reaches 99%, similar to the scenario when the network was trained on the original data from the MNIST dataset. This suggests that the features of source dataset are not distorted to the point in which the score degrades. It still maintains the resolution of the original MNIST. We also show the performance of the DANN approach mentioned earlier for comparison. This method reaches 90% accuracy on the colored dataset by minimizing the domain gap during the adversarial training. The proposed DDA method operates in the data domain and reaches an accuracy of more than 70% for inference on the target MNIST-M dataset. Meaning that the DDA might be integrated into the existing training routines without changing the network architecture. Finally, we note that the classification accuracy score reached by DDA is higher than what we would obtain without the proposed transformation of 30% (Figure 6).

### 3.4. Analysis

**Figure 7:**
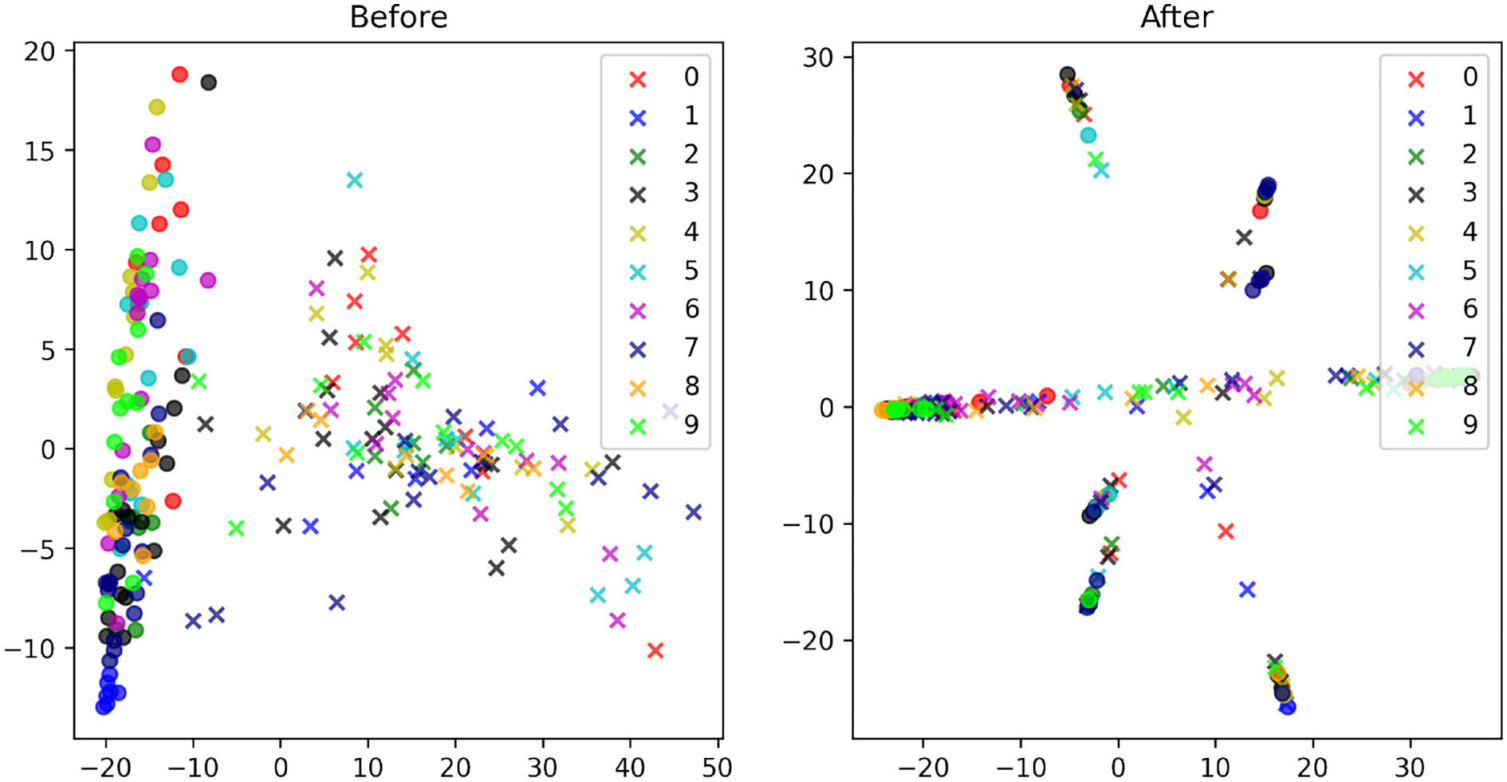
The PCA plots of the MNIST (circles) and MNIST-M (crosses) images with the corresponding digits they represent given in a unique color before **(left)** and after **(right)** the proposed transformations.

**Figure 8:**
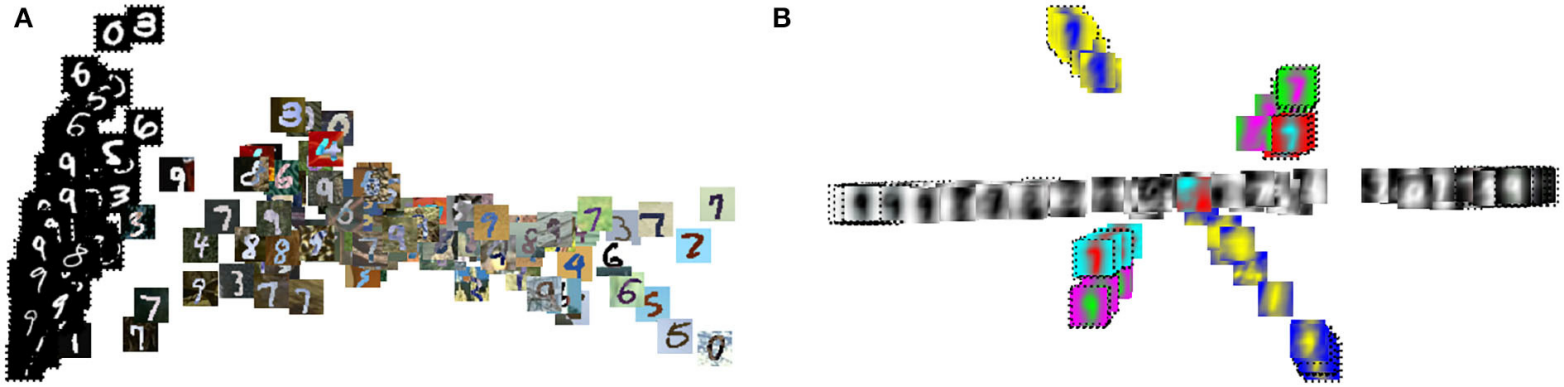
The PCA result of Figure 7 with the input images shown **(A)** before and **(B)** after the proposed transformation.

To further analyze the proposed direct domain adaptation (DDA) approach, we display the principal component analysis (PCA) for the source and target input features before and after the transformations 3 and 6 (Figure 7). Prior to DDA, the source (crosses) and the target (circles) input features occupy two different areas and they are separated mainly by the first principal component (the horizontal axis), which accounts for mainly the background of the images. After DDA, the source and target input features reasonably overlap and they follow certain slopes that are controlled by features in the images. A closer look is provided by Figures 8A,B in which the actual images before and after DDA are shown, respectively, as a function of the principal components. Along these slopes, which are linear combinations of the two principal components (eigenvectors corresponding to the two largest eigenvalues), we notice the images from the source and target data having, more or less, similar background. The source images (dashed box in Figure 8B) gravitate toward the edges of the slopes, while target images dominate the middle. In other words, the source images after DDA have larger magnitude of the principal components, or in other words, more pronounced features along the principal components, which could be helpful for the training.

**Figure 9:**
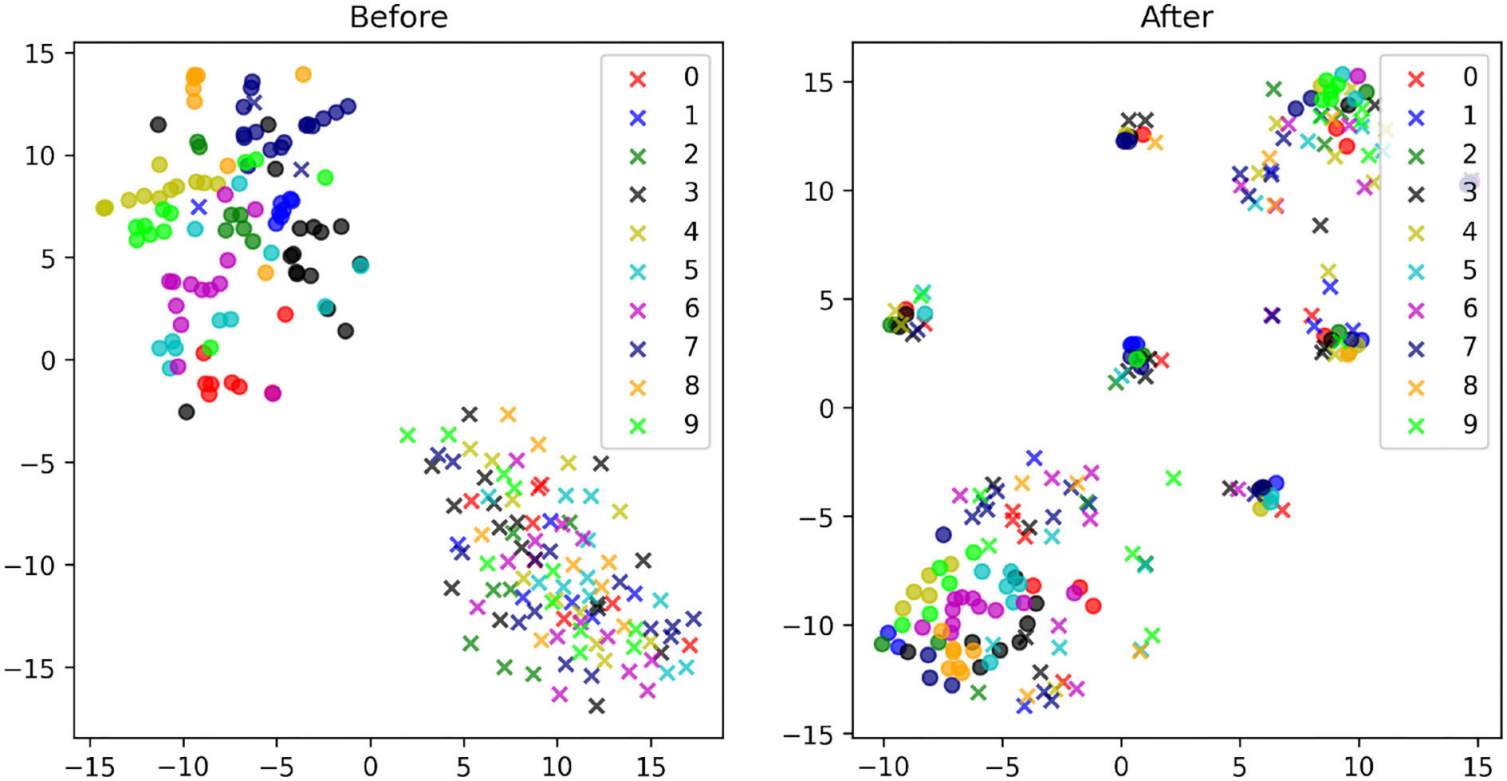
The t-SNE plots of the MNIST (circles) and MNIST-M (crosses) images with the corresponding digits they represent given in a unique color before **(left)** and after **(right)** the proposed transformations.

**Figure 10:**
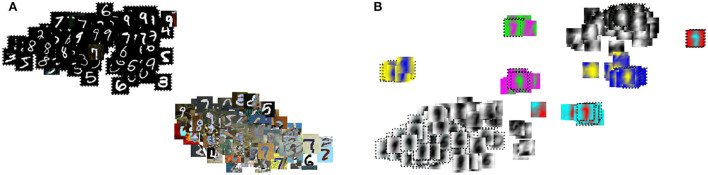
The t-SNE result of Figure 9 with the input images shown **(A)** before and **(B)** after the proposed transformation.

We further analyze the effect of the transformation using t-SNE, which exposes non-linear features in the images. We first compare the t-SNE for the MNIST (source) and MNIST-M (target) images before and after DDA (Figure 9). The separation between the two domains are more obvious in this plot. Thus, an NN model trained on the source data will have a hard time producing accurate classification on the target (MNIST-M) data due to the obvious covariate shift. This was reflected in the low 30% accuracy we got in applying the MNIST data trained model on the MNIST-M dataset. After applying DDA, we see clusters of mixed source and target data. A closeup look in Figures 10A,B reveals that these clusters are defined by the new general background of these images (which is a mix of the original backgrounds). Luckily, these clusters include both data domains. This implies that the transformation formed new shared backgrounds between both data (source and target), which explains the improved accuracy in the application of the trained model on the target data.

## 4. Discussions

The transforms proposed to the input features from the source and target data are linear, explicit, and efficient to apply. The mean of the auto-correlation (${\bar{a}}_{\text{s}}$, ${\bar{\text{a}}}_{t}$) and the mean of the randomly drawn pixel/pixels ($\hat{x_{s}}$, ${\hat{x}}_{t}$) can all be computed once and then applied to the input features like a linear layer. The Fourier domain provides a venue for an efficient application of these terms. Unlike conventional domain adaptation methods, the process here requires no training. Since these operations are linear, there is generally no loss in resolution in the input features, as these processes translate to multiplications in the Fourier domain. So the transformed input features carry more or less the same information as the original features. This assertion is supported by the training accuracy we achieved on the transformed MNIST data, which is at the same level as the original MNIST data using the same network.

Since DDA produces input features with equal amounts of source and target information, the distributions of the new transformed input features are expected to be closer. DDA also does not alter the general shape of the input feature, and thus, is expected to be applicable to not only classification problems, but also, segmentation, as well as other supervised learning tasks in general.

The mean of the auto-correlation of the input features is meant to extract the energy distribution information from one domain and insert it into the other domain through the convolution process. If the target domain includes a single example, the auto-correlation will contain more information of the energy distribution of that single example, and that information will be embedded in the training set. Recall that the auto correlation process admits zero phase, and thus, do not alter the general features of the input image. For example, if the target set only includes one sample given by the digit 7, the auto correlation will capture the background of this single image and the shape of this digit will be equally distributed on all the training set, and the differences given by the images of the digits will be maintained.

The PCA and t-SNE of the input features before and after DDA revealed to us that the approach managed to bring the input features (source and target) closer to each other, at least along the principal components. For the MNIST and MNIST-M data, the transformations produced a shared background for the input features that has elements from the original backgrounds of the source and target input features.

## 5. Conclusions

We proposed a direct and explicit technique to precondition the input features for a supervised neural network optimization so that the trained model works better on available label-free target data. This concept of direct domain adaptation (DDA) is based on incorporating as much information from the target input features into the training without harming the source input features crucial for the prediction. Considering the two domains (source and target), we specifically cross-correlate an input section from one domain with the mean of randomly picked samples from the same domain followed by a convolution with the mean of the auto-correlated sections from the other domain. For training the NN model, the input image is from the source domain, and for the application, the input image is from the target domain. The DDA operates in the data domain, and thus, does not require changes in existing network architecture and logic of the training workflow. Thus, it might be incorporated into existing solutions as a component of the data pre-processing routine. A test of this approach on the MNIST data as source and the MNIST-M data as target admitted 70% accuracy on the target data.

## Data availability statement

The datasets presented in this study can be found in an online repository. The repository can be accessed through the following link: https://github.com/swag-kaust/dda_pytorch.

## Author contributions

TA developed the idea and the methodology. OO ran and tested most of the examples and participated in preparing the manuscript. All authors contributed to the article and approved the submitted version.

## Conflict of interest

The authors declare that the research was conducted in the absence of any commercial or financial relationships that could be construed as a potential conflict of interest.

## Publisher's note

All claims expressed in this article are solely those of the authors and do not necessarily represent those of their affiliated organizations, or those of the publisher, the editors and the reviewers. Any product that may be evaluated in this article, or claim that may be made by its manufacturer, is not guaranteed or endorsed by the publisher.
